# The Status of Childhood Lead Poisoning and Prevention in Nevada, USA

**DOI:** 10.1100/tsw.2007.95

**Published:** 2007-03-30

**Authors:** Anne M. Rothweiler, Elena E. Cabb, Shawn L. Gerstenberger

**Affiliations:** University of Nevada Las Vegas, Department of Environmental and Occupational Health, 4505 Maryland Parkway Box 453064, Las Vegas, Nevada 89154-3064, USA

**Keywords:** lead, children, Medicaid, lead-based paint, minorities

## Abstract

One of the first steps in addressing the problem of childhood lead poisoning is to identify the possible sources of exposure in specific communities and target high-risk populations with appropriate interventions. Due to several factors, such as lack of funding and lack of blood lead reporting, little information exists regarding the occurrence of childhood lead poisoning and the prevalence of potential exposure sources in the state of Nevada. Following the recent establishment of a Nevada-based Lead Poisoning Program, we compiled the most current information available on Nevadans, and use this knowledge to suggest future research objectives and outreach activities for the state. Accordingly, we identify the characteristics of the vulnerable Nevada populations, explore possible sources of lead exposure unique to Nevada, and summarize the existing data on childhood lead poisoning. Emerging data indicates that Nevada is an area of rapid population growth, characterized by increasing immigration from Latin America, increasing numbers of children from low-income families with no health insurance. Also, childhood lead poisoning may arise from exposure to non-paint sources of lead. After presenting the Nevada statistics, we propose and recommend a set of research and outreach strategies that best suit the needs of Nevada residents.

## INTRODUCTION

Humans have known about the potential dangers of lead poisoning for centuries, and various countries have initiated programs and policies to prevent and treat illnesses associated with lead. High doses of lead can lead to seizures, coma, even death, but these exposures are rare[1]. The general population’s exposure to lead occurs at much smaller concentrations, with children being particularly susceptible to the adverse health effects of lead. Doses of lead below 10 ug/dL in children can lead to intellectual, behavioral, and developmental problems[2]. Research provides evidence of relationships between lead exposure at low doses and poor performance in school, loss of IQ, attention deficits, and juvenile delinquency, suggesting that there is no safe threshold for lead[3]. Children younger than two years exhibit behaviors that make them more susceptible to lead poisoning, as they spend more time on the floor where dust accumulates, and they display frequent hand-to-mouth activity, ingesting dust[4]. Once lead enters the body, it deposits in bone, and lead removal from the body is slower in children. An elevated blood lead concentration (10

ug/dL or more) may take months, even years, to decrease [1]. Because lead damage to a child may be permanent and can occur at low levels, prevention of lead exposure becomes a critical need and the most viable option in eliminating childhood lead poisoning.

In the U.S., childhood lead poisoning has decreased dramatically since the implementation of regulatory policies regarding lead paint, leaded fuels, and lead plumbing materials in the early 1970s, yet lead poisoning continues to affect children in the U.S.[1]. In 2002, approximately 300,000 children less than five years of age were lead poisoned[1]. According to 2004 CDC surveillance data, approximately 12% of U.S. children less than six years old received screening for blood lead levels, while almost 2% (over 42,000 children) had confirmed elevated blood lead levels greater than 10 ug/dL[5]. Based on the most current CDC data, only four states did not report blood lead levels to the CDC. These included Arkansas, Idaho, Nevada, and South Dakota[5].

Despite large amounts of national data on lead and childhood lead poisoning, the current status of lead exposure and childhood lead poisoning in Nevada is unknown. Due to inadequate funding, minimal blood lead testing, and the lack of mandatory reporting, lead has not received much attention in the state. The lack of mandatory blood lead reporting in Nevada has severely hindered effective monitoring or surveillance of lead in the human population. Consequently, this lack of data has created a perception that Nevada has no serious lead problems. In southern Nevada, the Southern Nevada Health District (SNHD) has worked to acquire voluntary data on blood lead levels in children and adults from several laboratories. These data demonstrate the presence of elevated blood lead levels and a need for more focused research in this area[6].

Without key information regarding pediatric lead poisoning, prevention, treatment, and education activities may not be as effective in reaching target populations. Accurate and current information regarding children with elevated blood lead levels in Nevada is an essential first step in properly addressing this public health issue. This paper summarizes the potential factors influencing lead exposure and lead poisonings in Nevada, and examines current information from local, state and federal sources that specifically identified lead related issues in Nevada. This examination will begin with a review of the regulatory agencies involved with lead issues, followed by an exploration of the characteristics of Nevadans, their environment, and will identify potential contributors to lead poisoning.

## Lead Regulatory Agencies

Federal, state, and local agencies established regulations and policies to protect adults and children from the adverse effects of lead poisoning. Several federal agencies maintain responsibilities in protecting humans from lead exposure. In 1988, the Lead Contamination Control Act authorized the CDC to develop programs to eliminate childhood lead poisoning. The CDC's Childhood Poisoning Prevention Branch was formed following this Act, and provides recommendations to states for eliminating lead poisoning. These recommendations include the development of plans to identify childhood exposures to lead and a method to conduct blood screening[1]. The U.S. Environmental Protection Agency (EPA) sets standards for lead levels in air, drinking water, dust, and soil. In addition, the EPA publishes information on lead and lead-based paint hazards, lead poisoning, and lead-based paint abatement[1]. The U.S. Food and Drug Administration (FDA) determines which ingredients and packing materials contain potential risks for lead exposure, while the U.S. Occupational Safety and Health Administration (OSHA) limits the concentrations of lead in air in the workplace[1]. The U.S. Department of Housing and Urban Development (HUD) establishes requirements for lead-based paint testing in federally funded housing and renovations in Public and Indian housing[1]. The Health Care Financing Administration (HCFA), within the U.S. Department of Health and Human Services, requires that state Medicaid agencies perform blood lead screenings on children enrollees[7]. Screenings must be conducted on enrollees at 12 and 24 months, and between 36 and 72 months if no prior screening was completed. Medicaid is a state-administered program that provides medical care to eligible low-income families and individuals. A

summary of the responsible agencies involved in lead regulation, the media they are affiliated with, and the current regulatory standards are shown as Table 1.

**Table 1**
**Federal Agencies and Regulations Regarding Lead in the Environment**

| Agency | Media | Level | Regulation or Recommendation | Reference |
|---|---|---|---|---|
| Centers for Disease Control and Prevention (CDC) | Blood | 10 ug/dL | • Action level for children<br>• Recommendation that states develop a method of identifying and testing lead poisoned children<br>• Recommendation that states test blood lead levels in children<br>• Childhood Lead Poisoning Prevention Program – eliminate childhood lead poisoning through policies, education, funding, and research | [8] |
| Consumer Product Safety Commission (CPSC) | Paints and Surface Coatings | 600 ppm | • Defines lead-containing paint<br>• Except for motor vehicles and boats applications, if a paint exceeding this limit is applied to an item intended for consumer use, the item becomes “banned hazardous products” | 16 CFR 1303 |
| Environmental Protection Agency (EPA) | Air | 1.5 ug/m$^3$ | • National Ambient Air Quality Standard | 40 CFR 50.12 |
| EPA | Water | 15 ug/L | • Action level for public drinking water | 40 CFR 141 |
| EPA | Dust - Floors | 40 ug/ft$^2$ | • Residential Lead Hazard Standard | 40 CFR 745.65 |
| EPA | Dust – Interior Window Sills | 250 ug/ft$^2$ | • Residential Lead Hazard Standard | 40 CFR 745.65 |
| EPA | Residential Bare Soil – Children’s Play Area | 400 ppm | • Residential Lead Hazard Standard | 40 CFR 745.65 |
| EPA | Residential Bare Soil – Non Play Areas | 1,200 ppm | • Residential Lead Hazard Standard | 40 CFR 745.65 |
| EPA | Lead-Based Paint Hazards | > 10 ft$^2$ deteriorated paint (exterior surface) or > 2 ft$^2$ deteriorated paint (interior surface) | • Definition of paint in “fair” or “poor” condition | 40 CFR 745 |
| Food and Drug Administration (FDA) | Food and Packaging | Various – 0.1 to 10 ppm | • Action levels for foods<br>• Handles ingredients and packaging materials | 21 CFR 184 |
| FDA | Fruit Beverages | 80 ug/kg | • Action Level | [9] |
| FDA | Foods packaged in Lead-Soldered Cans | 250 ug/kg | • Action Level | [9] |
| FDA | Ceramic Flatware | 3.0 ug/mL | • Action level | [9] |
| FDA | Ceramic Hollowware (Small) | 2.0 ug/mL | • Action Level | [9] |

**Table 1 cont.**

| | | | | |
|---|---|---|---|---|
| FDA | Ceramic Hollowware (Large) | 1.0 ug/mL | • Action Level | [9] |
| FDA | Ceramic Cups, Mugs, and Pitchers | 0.5 ug/mL | • Action Level | [9] |
| FDA | Bottled Drinking Water | 0.005 mg/L | • Action Level | 21 CFR 165.110 |
| Health Care Financing Administration (HCFA) | Blood | Test children at 12 and 24 months | • Within the Department of Health and Human Services<br>• Administers Medicaid program<br>• Requires state agencies to conduct blood lead screening | 42 CFR 1001 |
| HCFA | Lead-Based Paint | | • Requires paint testing in federally funded housing | 24 CFR 35 |
| Department of Housing and Urban Development (HUD) | Lead-Based Paint | | • Use of lead-based paint in residential structures built or rehabilitated by a federal agency or with federal assistance prohibited | 24 CFR 35 |
| HUD | Lead-Based Paint | 0.06% lead by weight in nonvolatile content | • Definition of lead-based paint manufactured after June 22, 1977 | 24 CFR 35 |
| Occupational Safety and Health Administration (OSHA) | Blood | 40 ug/dL<br>50 ug/dL | • In occupational exposures, action level - written notification and exam<br>• Removal of employee from exposure | 29 CFR 1910.1025 |
| OSHA | Air | 50 ug/m$^3$<br>30 ug/m$^3$ | • The Permissible Exposure Limit (8-hour average) in the workplace<br>• Action level | 29 CFR 1910.1025 |

Modified from: ATSDR, 2005

At the state level, the Nevada State Health Division has primacy regarding public health issues. Three counties in Nevada, Carson, Clark, and Washoe, have county health authorities. The 14 other counties, which are mostly rural, receive public health services directly from the state. Clark County has a Lead Poisoning Surveillance Program, and recently received a CDC grant to develop a Childhood Lead Poisoning and Prevention Program. Most states administer childhood lead poisoning prevention programs, gather information, and track data to measure program effectiveness. Characteristics such as population dynamics, immigration, age, race, ethnicity, income, Medicaid enrollment, and age of housing are reviewed. In addition, surveillance accumulates data on factors such as remodelling and renovation activities, urban or rural residences, nutrition, and parental occupations. Data collection enables reporting of number of children and adults tested, the number of elevated blood lead levels (EBLLs), the housing units with high risks of lead exposure, and summaries of lead poisoning cases. A review of this information should facilitate identification of high-risk populations for lead exposure.

The National Health and Nutrition Examination Survey (NHANES) tracks medical data and provide surveillance information. The NHANES-III data reported a nationwide decrease in blood lead levels[10]. Use of the national mean gives the impression that lead is not a problem, yet closer examination of the data suggests the existence of pockets of concern in specific populations. For instance, high-risk groups are derived from children younger than six years of age, minority children, inner-city urban areas, and children from low household income[10]. In order to determine if such high-risk groups exist in Nevada, an investigation into the population, demographics, income, housing, lead sources, and initial blood screening data was completed.

## Cultural Geography of Nevada

The state of Nevada occupies over 110,000 square miles, making it the seventh largest state in the U.S.[11]. Elevation ranges from 13,140 to 479 feet, with a mean elevation of 5,500 feet[11]. Deserts and mountains characterize Nevada's geography. This arid land lies within the Great Basin of the Basin and Range region. Sagebrush and creosote dominate the desert vegetation, and the mountain landscape includes over 150 ranges running north and south[11]. Between the ranges lie buttes, mesas, and alkali flats. Along the western edge of the state is the Sierra Nevada mountain chain, and the glacial Lake Tahoe occupies a valley in this region. Temperatures range from below freezing in the winter to over 120 degrees F in the summer[11]. Average precipitation in southern Nevada is four inches, while mountain ranges receive 20 to 30 inches in a year[11].

The largest city in Nevada is Las Vegas, located in the southern tip of the state. Reno is the second largest city. Carson City is the state capitol. The rest of the state's population resides in small towns and rural areas. Almost 80% of Nevada land is federally owned[11].

Nevada's economy is based on tourism, especially the gaming and resort industries. Centered primarily in Las Vegas, with additional hubs in Reno and Lake Tahoe, gaming taxes are the major source of state revenue[11]. The service sector occupies 50% of the employees living in Nevada[11]. In addition to gaming, Nevada's economy includes mining and industry. Nevada is the nation's leading producer of gold, silver, and mercury. Items such as gaming machines and products, aerospace equipment, lawn and garden irrigation devices, and seismic monitoring equipment are manufactured. Also, warehousing and trucking constitute a portion of Nevada's industries[11].

## Population

Nevada is experiencing extremely rapid population growth. For example, from 1990 to 2000, the population of Nevada increased by 66%, and continues to expand rapidly[12,13]. Between 1990 and 2000, the population of Las Vegas increased by 83%, making it the fastest growing metropolitan area in the U.S.[13]. Clark County has the largest population in the state, at almost 1.7 million people or 70% of the total Nevada population, and the largest city, Las Vegas, in the state. Certain populations are more sensitive to adverse health effects of lead exposure, specifically young children under the age of six years[1]. According to 2004 U.S. Census data, children under the age of five years comprise about 7.6%, (over 124,000), of the population of Clark County, and the population is projected to increase to over 180,000 by 2010[14,15].

The rapid growth pattern in Nevada, especially in Clark County, strains state and county resources, complicates data tracking, and prevents the state from keeping up with public health demands. Due to constrained fiscal conditions, the state struggles to create new programs and provide support for older programs[16]. As a result, lead research and surveillance has been minimal in Nevada. Due to limited resources, reporting, inspecting lead-contaminated housing, and training and outreach regarding lead poisoning threats, prevention, remediation, and safe work practices to the general public have not occurred at significant levels.

Another factor contributing to poor lead surveillance in Nevada may be the lack of primary care providers in the state[16]. The number of doctors cannot keep pace with the growing population, which may reduce the number of patients receiving blood lead screening and information about lead hazards and exposure. A difficulty in tracking the population arises with the drastic increases in population, compounded with a small number of physicians.

## Demographics

A major portion of Nevada's population growth comes from immigration. The population of Clark County continually diversifies, especially among Asians and Pacific Islanders, African Americans, Native Americans, and Hispanics. For example, minorities constitute 39% of the population of Clark County[17,18]. People of Hispanic origin, especially Mexico constitute the largest, and fastest growing, minority group, increasing by 220% during the 1990s in Las Vegas[17]. In 2000, the Hispanic population in Nevada equalled 20% of the total population; in 2006, approximately 26% of Clark County's population was Hispanic[17,18].

The population of another large minority group has increased in Nevada. People of Asian origin comprise 6% of the Nevada population, greater than the U.S. 4% national average[19]. The Asian population increased by 205% during the 1990s in Las Vegas[15]. Over 18% of the southern Nevada population is foreign-born, increasing from 10% in the 1990s [20]. A surge of immigration has occurred, and population estimates indicate that around 40% of the foreign-born population in Clark County is illegal, most moving from Latin America[20]. Approximately 23% of foreign-born people come from Asia, 49% come from Mexico, and 13% come from other Latin American countries[20].

This demographic pattern presents concerns for public health initiatives and prevention of lead exposure. Illegal immigrants often do not have medical insurance, occupy older housing, earn lower wages, and suffer prior exposure to lead[21]. For example, a study of pregnant women in South Central Los Angeles showed immigrant women had much higher blood lead levels than non-immigrant women. The study found that blood lead levels decreased as time spent in the U.S. increased[21].

## Income

One consequence of a large population influx involves an increase in the number of people living in poverty. The number of Nevadans living below the poverty level is increasing. In 1999, approximately 10% of Nevadans lived below the poverty level, and the percentage increased to 11% by 2003[22]. Estimates for Nevada households in 1999 indicate that the poverty rate for married couples with children under the age of five years was 6%, the rate for male heads of households with no spouse was 19%, and the rate for female heads of household with no spouse was 41%[22]. Access to medical insurance can be used as a proxy for poverty. Families with low income may not be able to afford insurance. Therefore, the amount of uninsured provides an estimate of the number of people unable to afford insurance, and unlikely to receive preventive medical care and blood lead screening. As a result, as the population of uninsured increases, so does the number of unscreened children and adults.

Nationally, the number of children less than 18 years of age without health insurance is increasing. In 2005, 11.2% of children in the U.S. did not have health insurance, while 19.0% of children under 18 years living in poverty did not have health insurance[23,24]. Over 19% of Nevadans lacked health insurance in 2003[12,23]. About 17% of Clark County residents in 2004 did not have medical insurance, and that percentage is increasing. In 2003, over 17% of children in Nevada were uninsured, compared to the national average of 12%[23]. According to a 2004 report produced by the Great Basin. Primary Care Association, over 74,000 children, (0 to 9 years), were uninsured in Nevada, with over 56,000 in Clark County alone. Children under the age of four comprise 40,679 uninsured people in the state, and 31,352 in Clark County, or 25% of all children less than four years in Clark County[26].

A closer examination of the Hispanic population shows a large portion is living in poverty conditions. The poverty rate of the Hispanic population in the U.S. in 2000 was 23%[22]. Poverty among Hispanic children increased by 251%[26]. Not only are Hispanics more likely to live in poverty, they are more likely to be uninsured[23]. In 2005, 21.9% of Hispanic children under 18 years in the U.S. did not have health insurance, compared to 7.2% among White children[23,24]. In 2001, 36% of Hispanics in Nevada did not have medical insurance[23]. Over 100,000 Hispanics in Clark County, (132,000 in Nevada), did not have medical insurance[25]. Also, Hispanics are three times less likely to have health insurance [15].

Another factor associated with income is enrollment in Medicaid. Of the 172,000 Medicaid enrollees in Nevada, 73% are families, children, and pregnant women[16,27]. Because Medicaid programs require blood lead screening, this group constitutes a large proportion of lower-income children able to receive screening free of charge. Due to a lack of information regarding childhood blood lead screening, it is difficult to determine how many children in Nevada's Medicaid programs received testing. According to information acquired from the Nevada State Health Division's EPSDT screening program, of the 66,247 children eligible for EPSDT screening, only 296 Medicaid recipients between the ages of zero and six years were tested during the 2004 fiscal year (Personal Communication with Nevada State Health Division).

Income levels also predict the status of several characteristics influencing blood lead levels. An NHANES study reports that children from poor families are eight times more likely to be poisoned than children from higher-income families[28]. Children covered by Medicaid are three times more likely to have elevated blood lead levels than children not under Medicaid[29]. Access to medical insurance and enrollment in Medicaid determine if children and families will receive information regarding lead toxicity and blood lead screening. Also, evidence supports a relationship between income level and age and maintenance of housing. Lower-income families are at a greater risk of lead exposure via deteriorating housing conditions[30].

## Housing

Along with an increase in poverty, rapid population growth can result in an increase in the number of people living in poor housing conditions. Poor housing conditions may increase the risks of lead poisoning. The Alliance for Healthy Homes offers criteria to define distressed or marginal housing, which may pose the most serious threat of lead poisoning[30]. Distressed housing may be defined through housing age, physical conditions, household income, and housing cost burden[30]. Older homes may contain more lead-based paint hazards than newer homes. Lead-based paint was banned in 1978, therefore a large percentage of pre-1980 homes (approximately 83%) contain lead-based paint[30]. Also, several industrial changes in the 1950s affected the lead content in paint. Paint manufacturers switched to latex paint and titanium dioxide pigment in oil-based paints[30]. Therefore, pre-1950 homes have higher concentrations of lead than later buildings. Second, several variables describe the physical conditions of a home. Indicators of potential lead exposure include deteriorating and/or peeling paint, cracked plaster, leaks, faulty plumbing and/or electrical systems, and lack of maintenance[30]. Third, the income of a household provides a surrogate measure of the amount of money spent on housing. Low-income is defined as less than 130% of the Federal poverty level[30]. The poverty level, as defined by the Office of Management and Budget and the U.S. Census Bureau, is calculated by comparing a family's income to a poverty threshold[24]. If the family income is less than the threshold, the family is considered to be in poverty[24]. The poverty threshold depends on the family size and composition. For example, the threshold value for a family consisting of two adults under 65 years and two children under 18 years is $19,806. Finally, the housing cost burden of a family represents the portion of income devoted to paying monthly housing costs, such as rent, mortgage, taxes, and utilities [30]. If the housing cost burden is high, (more than 50% of income), the family cannot afford to make repairs, perform lead hazard controls, or move to a better quality home[30].

Despite the ban in the 1970s, homes with lead-based paint still pose a potential source of lead exposure. Studies indicate that 25% of all U.S. housing has significant lead-based paint hazards [31]. A study of the housing stock provides information on several factors related to lead exposure. Rental properties are more likely to have lead-based paint than owner-occupied[31]. Low-income households are more likely to have lead-based paint hazards than middle and upper incomes[31]. Also, age of housing is an important variable. Pre-1960 housing units are five to eight times more likely to have lead-based paint hazards than units built from 1960 to 1978[31].

Nationally, many older homes exist. In 2000, 15% of the total housing stock in the U.S. was built before 1940[32]. For comparison, 8% of the housing stock was built between 1995 and 2000[32]. Although Nevada has many housing units, most were not built before 1978. From 1990 to 2000, a 60% increase occurred in the total housing units in Nevada, the largest increase of any state in the U.S.[32,33]. Over 26% of Nevada homes were built between 1995 and 2000, and 2% were built before 1940[32].

Although the majority of housing units in Nevada were built after 1980 (the median year of owner-occupied units is 1991, the median year of rental units is 1986), a large proportion of units may pose a risk of lead exposure through lead-based paint[15]. In 2000, Clark County had almost 600,000 total housing units[15]. Of the total housing units, approximately 27% were housing units built before 1979. Over 27,000 housing units were built before 1960[15]. Home investigations have been completed on approximately 400 housing units by a lead inspector for Clark County. Thus, formal sampling and investigations and risk assessments have not been performed on most housing units built before 1960.

Because older housing is more affordable, people in lower income groups often occupy them. According to a study of Las Vegas, North Las Vegas, Mesquite, and Boulder City, of the 35,775 housing units with potential lead-based paint hazards, 10,441 or 29% were occupied by low and extremely low-income families[34]. The combination of variables such as low income and uninsured families living in older homes increases the potential risks of lead exposure for children. Older homes have a greater chance of having lead-based paint, therefore lead exposure from deteriorating paint in pre-1979 homes may occur. If the occupants do not have insurance, they may not receive blood lead screening and unknowingly continue to be poisoned in their homes.

Marginal and distressed housing are two categories that HUD uses to focus on low-income families and their possible risk for lead exposure[32]. HUD defines these two categories based on: housing age, physical condition, household income, and housing cost burden. The predictors of marginal and distressed housing are useful in identifying the degree of risk for lead exposure. Often families with lower income have adequate housing but lack additional financial resources for repair and maintenance thereby allowing the home to fall into a severely inadequate housing status, which increases the risk of lead exposure[30]. For instance, older homes deteriorate at a faster rate then new homes and paint deterioration may occur. If household income barely covers the rent or mortgage, then the costs of repainting the home or repairing cracked and chipping paint is not feasible for the family. Lead hazards that were not present at the time of move-in may develop at a later time, as deteriorating housing conditions increase exposure to lead through dust and chips. Additional lead hazards include not only paint chips but also dust and contaminated soil around the home as a result of deteriorating paint. Exposure to children may occur through ingesting paint chips or soil (through hand-to-mouth contact) and inhaling contaminated dust[31]. Lead-based paint can contaminate a housing unit when the paint deteriorates, or it is disturbed during maintenance, repainting, remodelling, demolition, or removal of paint[31]. Homes with deteriorated paint are more likely to have increased levels of lead in dust inside, and in soil outside[31].

Information from the 2000 U.S. Census provides evidence as to the correlations between income, housing, and potential lead exposure. For example, homeowners more often live in new homes than renters, and owners have higher incomes than renters[32]. Income levels were higher for families living in new housing units, and lower for those living in old units[32]. Also, the U.S. Census Bureau measures crowding in housing units. A “crowded” unit has more than one person per room, and “severely crowded” has more than 1.5 persons per room. The Census data indicates that crowded households have lower income levels[32]. In Nevada, 9% of units are designated crowded, and 4% are severely crowded [32]. A high proportion of minority groups live in crowded conditions. In the U.S., 29% of Hispanic households are crowded, and 17% are severely crowded[32]. In comparison, the proportion of white, non-Hispanic households in crowded conditions was 2%, while severely crowded conditions comprised 0.6% of the households[32]. The foreign-born population in the U.S. constitutes a large portion of those living in crowded conditions. Almost 52% of all crowded units are occupied by foreign-born households[32]. Finally, an examination of housing structures and the number of units per structure can indicate potential areas of lead exposure. Structures with five or more units tended to have households with lower incomes than single-family homes and buildings with fewer units[32]. In Nevada, 23% of housing structures had five or more units[32].

A review of the information regarding housing highlights several key variables regarding lead exposure. These data suggest a focus on older, crowded homes, and low-income households. Also, minority renters in structures with more than five units may be at increased risk of lead exposure.

## Sources of Lead Exposure

National research indicates that common sources of lead exposure to humans in the U.S. involve older homes, with lead-based paint and old leaded pipes. Because Nevada has a smaller housing stock of pre-1978 homes, older homes may not be a major source of lead in the state. Other major sources of human lead exposure include lead gasoline additives, food can soldering, ceramic glazes, drinking water systems, and cosmetic and folk remedies[35]. Hydrologic characteristics of eastern states, such as acidic or soft waters, increase the solubility and presence of lead from old pipes in water[1]. In Nevada, water is characterized by more basic and hard water qualities. Therefore, lead exposure through drinking water may not be a problem in Nevada. Exposure to lead through soil near roadways, industrial complexes, power plants, landfills, hazardous waste sites, mining areas, incinerators, and old orchards are major pathways at the national level. Again, the characteristics of Nevada and the placement of these facilities require further research as potential sources of lead exposure.

## Non-Paint Sources

Various environmental sources related to cultural practices have been implicated in EBLL cases[34]. Researchers have found evidence of lead poisoning causative agents that includes Mexican candies, folk remedies, imported pottery and ceramics, pewter key chains, and bathtub tiles[36,37,38]. Recent cases of childhood lead poisoning in the US have been attributed to lead contaminated Mexican candies[36,39,40]. The same trend is suspected in Nevada in cases where no apparent lead exposure is present in the home and Mexican candy consumption is confirmed. The Food and Drug Administration currently have no enforcement standards on imported candy products[41]. A significant lead hazard may be present in all components of Mexican imported candies from the tamarind or salt-based candy itself, sticks, straws, to associated wrappers and containers[39,40].

Many Hispanic populations practice traditions which incorporate homeopathic medicines for treating gastrointestinal illnesses, often called “empacho”[37,42]. The folk medicines are not FDA regulated and have been found to contain lead levels as high as 770,700 parts per million[36]. Folk remedies are usually recommended by healers and have names such as Greta and Azarcon[37]. Due to the nature of folk remedies, many limitations for further research arise. Parents are wary of disclosing information about the lead-based remedy, healers often work out of their homes, and different names for the remedies complicate research.

Family use of lead-glazed pottery has been associated with elevated blood lead levels[43]. Traditional ceramic cookware poses a threat to children who ingest food prepared or served in the lead-glazed ceramic bowls, plates, or bean pots. Lead leaching into food products or possible dust inhalation from broken lead-glazed pottery are two main hazards related to ceramic pottery[38]. Other unique sources of lead poisoning have been found throughout the U.S.[42,44]. Sources include apple cider, lead wicked candles, leaded fishing sinkers, miniblind weights, toy necklaces, spices, and bullet fragments[45,46,47,48,49]. Current home investigations in Nevada have identified sources of lead exposure such as pewter keychains, bathtub tiles, and products with leaded-ink (unpublished data).

## Blood Lead Screening

The state of Nevada does not have mandatory blood lead level reporting requirements. Consequently, minimal information can be found regarding blood lead levels in Nevada's population, very little investigative work has been completed on homes and families, and limited surveillance work has been recorded. Because of the lack of reporting, it is difficult to obtain accurate lead levels or determine the number of lead poisonings in the population. Despite these limitations, the Southern Nevada Health District has secured relationships with several laboratories that perform blood lead testing, and has acquired some voluntary information on blood lead levels in the state. These data, (see Tables 2 and 3), provide a cursory view of the current state of childhood blood lead concentrations. It should be noted that due to the current reporting structure, the information has been derived mainly from insured patients[6].

Of the 2,791 reports from 2004, 17 (0.6%) were elevated above 10 ug/dL[6]. The SNHD report shows a large portion of lead poisoning cases were in the Hispanic community. Of the 17 total EBLLs, 15 (88%) were Hispanic[6]. The percentage of children with elevated blood lead levels is small, but the portion of the total child population actually tested is also small. According to U.S. Census data, the population of children less than five years in Clark County was 124,000[15]. Thus, only 2% of the population of Nevada children was tested. The SNHD is currently developing a Childhood Lead Poisoning Prevention Program. As the number of reports increases, the number of EBLLs is likely to increase proportionately. With more data, the picture of lead in Nevada will become clearer.

**Table 2**
**Clark County, NV Blood Lead Levels in Children (August 2004 through August 2005)**

| | Non Detect (0 ug/dL) – LOD | 1 – 9 ug/dL* | EBLL (10+ ug/dL) | Total |
|---|---|---|---|---|
| Child | 2,153 (77%) | 621 (22%) | 17 (0.6%) | 2,791 |

Modified from Sands, 2005

**Table 3**
**Clark County, NV Blood Lead Levels in Children by Ethnicity (August 2004 through August 2005)**

| | Non Detect (0 ug/dL) – LOD | 1 – 9 ug/dL* | EBLL (10+ ug/dL) | Total |
|---|---|---|---|---|
| Hispanic Child | 1,094 (51%) | 312 (50%) | 15 (88%) | 1,421 |
| Non-Hispanic Child | 1,059 (49%) | 309 (50%) | 2 (12%) | 1,370 |
| Total Child | 2,153 | 621 | 17 | 2,791 |

Modified from Sands, 2005

*Due to the varying detection limits, (<1 and <3 ug/dL for two laboratories), the 1-9 ug/dL category may be underestimated.

## SUGGESTIONS FOR THE FUTURE

In order to more accurately address the issue of lead as a public health concern in Nevada, research efforts will need to recognize both national and local trends. A rapidly increasing population, with increasing diversity and a large proportion of Hispanic and illegal immigrants, characterize Nevada's current population growth. The amount of people living in poverty and without access to health insurance are also increasing. The housing stock built before 1979 remains relatively untested for lead-based paint hazards. Also, little is known about the prevalence of potential sources of lead exposure such as Mexican candies, ceramic and glazed pottery, and folk remedies in Nevada. Finally, limited data collection hampers efforts to identify, isolate, and eliminate lead exposure and lead poisonings in the state.

Establishment of comprehensive statewide screening, surveillance, environmental risk assessments, and primary prevention outreach and education programs would increase knowledge and prevention of lead poisoning. The program would need to gather information on the status of lead, initially focusing on the crucial components of blood lead screening and reporting EBLLs. Individuals identified with an EBLL should receive environmental follow-ups to investigate potential sources, and determine if removal and/or abatement or interim controls should be performed. Home investigations and risk assessments should be conducted, and should target pre-1978 homes, which are occupied by low-income families. In order to focus efforts and improvements, a program must develop the capacity to track, store, and analyze information. This information could then direct future decisions, actions, and policies. Specific programs, interventions, and educational measures could be created, and specific research strategies to define the local sources and activities resulting in lead exposure could be organized. Also, a program in Nevada needs to be able to research unique sources of lead for greater understanding of risk of exposure. Children may be exposed to multiple sources; therefore investigations need to encompass all possible sources of lead.

Increasing screening of child blood lead levels and improvements in reporting will increase understanding of the status of lead in Nevada. More detailed reporting would provide a better description of the most susceptible populations in Nevada. Currently, the blood lead data are grouped into two very broad population categories, Hispanic and Non-Hispanic. African-American communities typically have elevated blood lead levels also, but would be classified as non-Hispanic given the current reporting structure[1]. Expanding the criteria to match established federal categories of ethnicity would be a good first step.

Another area of future efforts involves targeting high-risk neighborhoods. Prevention and management activities could be focused on communities with older housing units, high levels of crowding, and low income. Also, minority communities, especially Hispanic and perhaps African-American and Asian, may be at higher risk of lead exposure. These communities may also have a large proportion of immigrants, who have prior exposures to lead. In order to increase understanding of the health hazards of lead, researchers and public health programs will need to work with the community to conduct screenings and home investigations. Because of traditional and cultural practices, home investigations and community-based outreach need to be established to identify and eliminate practices that put people at greater risk of lead exposure.

## CONCLUSION

Regulations, and the federal and state agencies responsible for lead policies, seek to eliminate childhood lead poisoning by 2010. The CDC and individual state health authorities have developed educational programs, and surveillance and investigative methods to determine, treat, and manage poisoning cases as well as report progress. Information from these programs indicates that certain characteristics may be used to predict, track, and eliminate potential lead exposures and poisonings. These variables include population, income, and access to medical insurance, housing, and exposure to non-paint lead sources.

Examinations of the population and environmental characteristics in Nevada clearly indicate a need for further research. Fortunately, these efforts have been initiated by the SNHD and the UNLV School of Public Health. Several conditions may increase lead exposure. The rapidly growing population stretches resources. The rapidly growing number of immigrants, especially of Hispanic origin, may have been exposed to high concentrations of lead before moving to the U.S. Many households live below the poverty level, and as a result, may not have access to medical care, or be able to afford newer housing, purchase newer housing, prevent overcrowding, or maintain housing to prevent deterioration of paint. Also, non-paint sources, such as glazed ceramics and folk remedies, may be used extensively.

Since lead poisoning symptoms may be subtle, those who spend the most time with children, such as parents, and those who monitor their health, such as physicians, are uniquely positioned to notice problems associated with lead. Yet, parents and physicians need to be armed with the information first to be effective. Through education, healthcare provider interventions, prevention, management, and treatment protocols, state legislation, and a focus on high-risk communities, childhood lead poisoning could be prevented and eliminated. Tracking these activities would describe the status of lead in Nevada and provide us with a benchmark to evaluate programs, as well as direct all future efforts. Indeed, investigation into the extent of lead exposure and into the specific and unique prevention, treatment, and management activities appropriate to Nevadans arises as a crucial step in protecting children from lead poisoning. A major first step in eliminating lead poisoning was the recent acquisition of a Childhood Lead Poisoning Prevention Program grant from the CDC, awarded to the SNHD. This grant provides much needed resources and personnel to tackle the numerous issues described herein. For example, as a part of the grant, UNLV has developed methods to identify lead poisoning problems in Nevada, and have established protocols for conducting home investigations.

## RECOMMENDATIONS

- Collect statistics on population growth, housing, income, access to medical care
- Collect information on minority communities and prevalence of cultural practices with potential for lead exposure
- Establish statewide screening and outreach
- Increase awareness of childhood lead poisoning and identify risk factors and appropriate control methods
- Inform and educate healthcare providers and physicians
- Increase information gathering activities by health care agencies and increase general knowledge among the population
- Establish mandatory reporting of blood lead levels and screening of children
- Improve data gathering and analysis
- Establish targeting criteria to investigate communities at greater risk of lead exposure

## ACKNOWLEDGEMENTS

We would like to thank our collaborators and friends at the Southern Nevada Health District, especially Keith Zupnik, Wilbert Townsend, Brian Labus, Brenda Argueta, Patricia Rowley, and Lawrence Sands.
